# The double-edged sword of autistic traits: opposing pathways to loneliness via friendship similarity in Chinese adults

**DOI:** 10.3389/fpsyg.2026.1873567

**Published:** 2026-06-18

**Authors:** Yun-chang Chen, Tian Wu

**Affiliations:** 1 School of Education Science, Nanjing Normal University, Nanjing, China; 2 College of Education Science and Technology, Nanjing University of Posts and Telecommunications, Nanjing, China; 3 Department of Rehabilitation Sciences, Nanjing Normal University of Special Education, Nanjing, China

**Keywords:** attention to detail, autistic traits, Chinese adults, interest similarity, life satisfaction, loneliness

## Abstract

**Background:**

Loneliness is a pervasive concern in the Broader Autism Phenotype, yet research often treats autistic traits as homogeneous risk factor, obscuring potential adaptive pathways. The distinct roles of social-communicative difficulties versus cognitive traits (e.g., Attention to Detail) in shaping friendship remain underexplored, particularly in non-Western contexts.

**Method:**

A sample of 1,076 Chinese adults completed the Autism Spectrum Quotient, adapted friendship similarity items (interest and personality similarity), the UCLA Loneliness Scale, and the Satisfaction with Life Scale. After excluding 4 participants with non-binary gender, the analytical sample was 1,072. Serial mediation models with 5,000 bootstrap resamples were employed to examine whether autistic trait dimensions indirectly predicted life satisfaction through interest similarity and loneliness.

**Results:**

Analyses revealed divergent pathways. Social skills (*β* = −0.105) and communication (*β* = −0.117) difficulties negatively predicted interest similarity, whereas Attention to Detail was a positive predictor (*β* = 0.059). Serial mediation confirmed that Attention to Detail had a significant positive indirect effect on life satisfaction via the interest similarity–loneliness chain [indirect effect = 0.00059, bias-corrected 95% CI (0.00006, 0.00159)], contrasting with the negative indirect effects of Social Skills (indirect effect = −0.00083) and Communication (indirect effect = −0.00123). A robustness check using personality similarity as mediator showed that the protective effect was specific to interest similarity.

**Conclusion:**

These findings challenge the monolithic deficit model by identifying Attention to Detail as a protective factor facilitating interest-based friendship connection. Distinguishing between social and cognitive autistic trait dimensions is crucial for developing tailoring, interest-based interventions to alleviate loneliness in Chinese adults.

## 1 Introduction

Autism spectrum disorder (ASD) is increasingly understood not as a categorical diagnosis but as a continuum of traits extending into the general population, a concept known as the Broader Autism Phenotype (BAP) (Baron-Cohen et al., 2001; Wheelwright et al., 2010). Individuals with elevated autistic traits (ATs) in non-clinical samples often exhibit challenges in social communication and rigid behavioral patterns similar to, though milder than, those in clinical ASD (Lamport and Zlomke, 2014; Stice and Lavner, 2019). Growing empirical evidence indicates that high levels of ATs are associated with poorer psychosocial outcomes, particularly increased loneliness and diminished life satisfaction (Suzuki et al., 2021). A recent meta-analysis further confirms that loneliness is both highly prevalent and clinically significant in autistic populations, with strong associations with anxiety and depression (Hymas et al., 2024). Moreover, social and nonsocial reward processing has been shown to moderate the relationship between autism symptoms and loneliness across both clinical and non-clinical groups (Han et al., 2019). While this negative association is well-documented (Grace et al., 2024), the precise interpersonal mechanisms underpinning it remain under-explored. Specifically, it is unclear how distinct dimensions of autistic traits (e.g., social vs. non-social) intersect with the qualitative morphology of friendship to differentially predict well-being.

Existing literature has established the multidimensional nature of ATs, highlighting that the divergence between social difficulties and non-social traits (e.g., attention to detail) manifests uniquely across different cultural contexts (Agelink van Rentergem et al., 2019; English et al., 2020). China’s cultural context, characterized by collectivism and social harmony (Chen and French, 2008), relies heavily on high-context communication where meaning is embedded in implicit relational cues (Gao et al., 1998). Consequently, social competence in China is not merely viewed as a functional skill but is often conflated with moral character (Ren) and interpersonal maturity (Zuo Ren) (Hwang Hwang, 1987). In such an environment, deconstructing ATs into these dimensions is therefore crucial to understanding their specific pathways to well-being in the Chinese context.

Defined by Peplau and Perlman (1982) as a gap between desired and actual relationships, loneliness is a central psychosocial concern in autism research (Grace et al., 2022). Importantly, contemporary research challenges the assumption that autistic individuals are socially unmotivated. Instead, many adults with high ATs report strong desires for connection (Jobe and White, 2007; Stice and Lavner, 2019) but experience a “Loneliness Paradox”: they may possess social networks yet suffer from a profound sense of disconnection (Müller et al., 2008).

To explain this, recent scholarship has shifted from deficit-based models to a relational mismatch perspective, often framed within the Double Empathy Problem (Milton, 2012). This theory posits that loneliness arises not solely from individual social deficits, but when an individual’s communication style and relational expectations are misaligned with those of their social environment (Crompton et al., 2020). Consequently, establishing supportive friendships serves as a crucial protective buffer; by providing a ‘relational niche’ of mutual understanding, perceived social support acts as a vital mediator in mitigating the sense of isolation resulting from such mismatches.

Friendship serves as a vital buffer against loneliness (Mazurek, 2014), providing opportunities for mutual understanding, emotional support and a sense of belonging (Jamil et al., 2017) but its protective role for individuals with high ATs is complex.

While traditional literature predominantly evaluates interpersonal well-being through the lens of friendship quality—encompassing dimensions such as emotional intimacy and perceived support—such frameworks may inadvertently pathologize neurodivergent relational styles by benchmarking them against neurotypical standards. In contrast, shifting the empirical focus to friendship similarity, or interpersonal homophily, allows for a non-deficit investigation into the shared cognitive and communicative resonances that underpin social bonding in the broader autism phenotype (BAP). This is particularly salient under the Double Empathy framework (Milton, 2012), which posits that communication friction arises not from individual impairment, but from a mismatch in relational disposition between individuals of differing neurotypes. Emerging evidence suggests that Perceived Similarity (or Homophily)—sharing interests, neurotypes, or ways of thinking—is a potent predictor of relational satisfaction for individuals with high Ats (Bolis et al., 2021). For this population, friendship forms that effectively alleviate loneliness may diverge from normative social expectations, placing greater emphasis on relational compatibility, shared interests, and interactional fit rather than emotional intensity or social frequency (Umut and Köse, 2025). Consequently, the specific quality and structure of friendships, rather than mere quantity, become critical determinants of loneliness (Feller et al., 2024).

To contextualize these dynamics pathways, loneliness within the BAP should be reframed through a holistic social connection framework (Holt-Lunstad, 2022, 2025), which conceptualizes social health as a complex ecosystem rather than a binary state of deficit. Within this architecture, interpersonal mitigation of loneliness does not rely exclusively on intensive, high-quality dyads; rather, the value of ‘weak ties’ and incidental social contacts, such as subcultural niche interactions, pivots as a critical buffer for individuals with high autistic traits (Pellicano and Heyworth, 2025). By capturing friendship similarity, our model taps into this broader ecosystem, demonstrating how shared cognitive processing styles create specific relational niches that foster social connection without demanding masking behavior.

Despite the growing consensus that ATs are multidimensional, the majority of existing research relies on total AQ scores or treats social and non-social traits as homogenous risk factors. This aggregation risks masking specific “adaptive” pathways within the BAP, particularly in non-Western contexts where cognitive strengths might buffer against social friction. Specifically, while the social-communicative difficulties are well-documented predictors of loneliness, the role of cognitive traits, such as Attention to Detail remains ambiguous.

To address this gap, the present study moves beyond global model fitting to conduct a fine-grained analysis of how distinct AT dimensions differentially predict friendship similarity and subsequent well-being among Chinese adults. We employ multivariate regression analyses with effect size visualization to directly contrast the magnitude and direction of social versus cognitive traits.

Based on the theoretical framework of the Double Empathy Problem and the relational mismatch perspective, we propose the following specific hypotheses:

*H1 (The Divergent Pathways Hypothesis)*: Autistic traits will demonstrate a multidimensional association with friendship similarity. Specifically, social-communicative dimensions (e.g., Social Skills, Communication) will be negative predictors of friendship similarity (functioning as risk factors), whereas the cognitive dimension of *Attention to Detail* will be a positive predictor of friendship similarity (functioning as a protective factor), reflecting a unique "cognitive advantage" in forming niche relational bonds.

*H2 (The Mediation Hypothesis)*: Friendship similarity will mediate the relationship between autistic traits and loneliness. We posit that the heightened loneliness observed in individuals with high social difficulties is partially explained by a lack of relational similarity (i.e., relational mismatch), rather than solely by social frequency.

*H3 (The Serial Mediation Hypothesis)*: There will be a significant serial mediation effect from autistic traits to life satisfaction through friendship similarity and loneliness (Autistic Traits → Friendship Similarity → Loneliness → Life Satisfaction). Crucially, we expect divergent indirect effects: social difficulties will impair life satisfaction through this chain, while *Attention to Detail* will enhance life satisfaction by facilitating greater friendship similarity and subsequently reducing loneliness

## 2 Methods

### 2.1 Participants and procedure

Participants were recruited via widely used social media platforms in China, including WeChat and QQ. A total of 1,076 adults completed the anonymous online questionnaire between June and October 2024 via a secure web-based survey platform (Survey Star).

Prior to the survey initiation, all participants provided electronic informed consent. They were fully briefed on the study’s purpose, the voluntary nature of their participation, and their right to withdraw at any time without penalty. The study protocol was approved by the Institutional Review Board of Nanjing Normal University of Special Education and adhered to the ethical standards of the Declaration of Helsinki. To ensure data validity, quality control questions were embedded within the survey; participants who failed these checks were excluded. The survey employed a forced-response design for key items to minimize missing data.

The survey employed a forced-response design for key items to minimize missing data. Participants self-reported their gender via a single categorical item with three options: Male (41.1%, *n* = 442), Female (58.6%, *n* = 630), and other (0.4%, *n* = 4). Because the item did not explicitly distinguish between biological sex (assigned at birth) and self-identified social gender, and given that the Other subgroup was too small for independent statistical inference, the 4 participants selecting Other were excluded from gender-stratified analyses but retained in all full-sample models. We note this as a limitation in Section 4.5 and recommend that future studies employ separate items for sex and gender identity in accordance with SAGER guidelines (Heidari et al., 2016).

Demographic characteristics of the full sample (*N* = 1,076) are presented in Table 1. The sample was predominantly young adults. The original age distribution included a small number of participants under 18 (*n* = 2) and over 40 (*n* = 2); to ensure robust group-level inference, these participants were merged with the adjacent age bands. Accordingly, for all subsequent analyses, age was dichotomized into 25 years and below (59.9%, *n* = 644; comprising the original 18–25 group and the 2 participants under 18) and 25–45 years (40.2%, *n* = 432; comprising the original 25–40 group and the 2 participants over 40). Educationally, the majority held or were pursuing an undergraduate degree (89.3%, *n* = 961), with smaller proportions at the master’s level (9.7%, *n* = 104) or doctoral level (1.0%, *n* = 11). In terms of academic discipline, participants were classified into STEM (Science, Technology, Engineering, and Mathematics; 63.7%, *n* = 685) and non-STEM fields (Humanities, Social Sciences, Arts, and other disciplines; 36.3%, *n* = 391). Because individuals in STEM fields have been shown to report higher systemizing traits and attention to detail (Baron-Cohen et al., 2001), discipline was included as a covariate to isolate the unique contribution of autistic traits to friendship similarity.

**Table 1:** Demographic characteristics of the sample (*n* = 1,076).

Variable	Category	*n*	%
Gender	Male	442	41.08
	Female	630	58.55
	Other	4	0.37
Age group	18–25 years	644	59.85
	25–40 years	432	40.15
Education	Undergraduate (incl. in progress)	961	89.31
	Master’s (incl. in progress)	104	9.67
	Doctoral (incl. in progress)	11	1.02
Academic discipline	STEM	685	63.66
	Non-STEM	391	36.34

*n* = 1,076. Gender was assessed via a single self‑report item (Male, Female, Other) and did not separately measure biological sex. Due to small cell size, participants selecting other (*n* = 4) were excluded from gender‑stratified analyses but retained in full‑sample models (analytical *n* = 1,072). The two participants under 18 were combined with the 18–25 group due to negligible cell size; the two participants over 40 were combined with the 25–40 group. For subsequent analyses, age was dichotomized into 25 years and below (*n* = 644) and 25–45 years (*n* = 432); education was collapsed into Undergraduate (*n* = 961) and Graduate (*n* = 115); discipline was dichotomized into STEM (*n* = 685) and non‑STEM (*n* = 391). STEM = Science, Technology, Engineering, and Mathematics.

Although this study investigated the broader autism phenotype (BAP) in a non-clinical community sample, the distribution of AQ total scores confirmed substantial representation of elevated autistic traits. On the continuous 1–4 scoring scheme (total score range: 50–200), the sample mean was 118.18 (SD = 12.13, range = 75–167). While the widely cited clinical cut-off scores of 26 or 32 pertain specifically to the binary (0–1) scoring method, and are not directly transferable to Likert scoring (Murray et al., 2017), we note that 13.0% of the sample (*n* = 140) scored more than one standard deviation above the mean, indicating a substantial upper-tail representation of elevated autistic traits and confirming the empirical relevance of our community sample.

### 2.2 Measures

#### 2.2.1 Autistic traits

Autistic traits were assessed using the Mandarin version of the 50-item Autism-Spectrum Quotient (AQ; Zhang et al., 2016). Items were rated on a 4-point Likert scale ranging from *definitely disagree* to *definitely agree*. Following standard practice, half of the items were reverse-scored so that higher scores consistently indicate greater endorsement of autistic traits. We focused on five theoretically established subscales: Social Skills, Attention Switching, attention to Detail, Communication, and Imagination. Internal consistency for the total scale was acceptable (Cronbach’s *α* = 0.76), consistent with the broad dimensionality of the BAP construct in general populations. However, the primary theoretical focus and analytical units of this study reside strictly on subscale-level variance. The Social Skills (*α* = 0.81), Communication (*α* = 0.64), and Attention to Detail (*α* = 0.60) subscales demonstrated adequate internal consistency. Although the reliability of Attention to Detail was somewhat lower, it closely mirrored values reported in both the original UK sample (*α* = 0.63) and the Hong Kong Chinese sample (*α* = 0.54), suggesting that this reflects the intrinsic content heterogeneity of the dimension—items range from noticing patterns to fascination with numbers—rather than measurement deficiency. These three subscales constituted the focal predictors in all primary analyses.

In contrast, Attention Switching (*α* = 0.47) and Imagination (*α* = 0.31) demonstrated poor internal consistency in the present sample. This is not unprecedented: Imagination showed variable reliability across cultural adaptations (α = 0.65 in the UK; not reported in the Hong Kong version), and Attention Switching was similarly lower in the Hong Kong sample (α = 0.60) than in the UK (α = 0.67). Given that these subscales fell below conventional reliability thresholds, they were combined into a composite “Other Cognitive Features” index (20 items; α = 0.59). This composite was used exclusively as a control variable in supplementary models and was not included in the primary serial mediation analyses.

All variance inflation factors (VIFs) among the three focal subscales were well below 2.0 (Social Skills = 1.85, Communication = 1.71, Attention to Detail = 1.14), confirming that multicollinearity did not distort the divergent pathway estimates.

#### 2.2.2 Friendship similarity

Friendship similarity was assessed using two single-item measures adapted from the Friendship Questionnaire (FQ; Baron-Cohen and Wheelwright, 2003). Participants rated their perceived similarity with friends in two distinct facets: personality similarity (“How similar are you and your friends in terms of personality?”; fq6) and interest similarity (“How similar are you and your friends in terms of interests?”; fq7). Both items were rated on a 4-point scale ranging from 1 (very different) to 4 (very similar), with higher scores indicating greater perceived homophily.

The two items showed a moderate inter-item correlation (*r* = 0.41, *p* < 0.001), indicating that they capture related but distinguishable facets of friendship similarity. Given that personality similarity and interest similarity are theoretically distinct under Systemizing Theory, and that aggregating them into a single score would obscure their potentially differential mechanisms, they were treated as separate single-item indicators. No internal consistency coefficient is reported for a two-item scale, as single-item measures inherently do not possess internal consistency reliability. Instead, their moderate correlation supports evaluating them as independent pathways.

In all primary serial mediation analyses, interest similarity (fq7) served as the core mediator. This choice was theoretically motivated: Systemizing Theory (Baron-Cohen, 2009) posits that individuals with a detail-oriented cognitive style preferentially seek out structured, rule-bound domains and may form social bonds through shared epistemic interests—such as mutual fascination with specific topics, technical hobbies, or analytical pursuits—rather than through broad socio-emotional fluency. Interest similarity is therefore the most theoretically relevant pathway through which Attention to Detail may facilitate social connection. Personality similarity (fq6) was retained in robustness checks to empirically test whether the protective pathway operates specifically through shared cognitive interests, or alternatively through generalized interpersonal compatibility. As detailed in Section 3.3, the protective indirect effect of Attention to Detail was significant only through interest similarity, not through personality similarity, supporting the specificity of the proposed mechanism.

#### 2.2.3 Friendship status

Participants reported their current friendship status via a categorical item asking whether they currently maintained close friendships (0 = Has close friends; 1 = No close friends). This served as a control variable to distinguish relationship quality from relationship presence.

#### 2.2.4 Loneliness

Loneliness was measured using the short-form UCLA Loneliness Scale (ULS-6) (Russell, 1996). The scale consists of six items (e.g., “I feel isolated from others”) rated on a 4-point Likert scale ranging from never to always. Item scores were averaged to compute a composite loneliness score, with higher values indicating greater subjective loneliness. The scale demonstrated good internal consistency in the present sample (Cronbach’s *α* = 0.85).

#### 2.2.5 Life satisfaction

Life satisfaction was assessed using the Satisfaction with Life Scale (SWLS) (Diener et al., 1985), a 5-item global measure of cognitive well-being. Items were rated on a 7-point Likert scale from strongly disagree to strongly agree. Item scores were averaged to produce a final life satisfaction score, with higher scores indicating greater well-being. The SWLS demonstrated high internal consistency (Cronbach’s *α* = 0.88).

### 2.3 Statistical analysis

Data analyses were conducted using Stata 17.0. First, descriptive statistics and Pearson correlations were computed to examine the initial relationships among variables, separately for the total sample and stratified by gender.

To disentangle the specific contributions of each autistic trait dimension, we conducted a hierarchical multiple regression analysis. Demographic covariates: gender (male = 1, female = 0), age group (25 years and below vs. 25–45 years), education (undergraduate vs. graduate), STEM discipline (STEM vs. non-STEM), and friendship status (has close friend vs. no close friend), were entered in the first block. The three focal AQ subscales (Social Skills, Communication, and Attention to Detail) along with the composite Other Cognitive Features index were entered in the second block. Variance inflation factors (VIFs) for all predictors were calculated to ensure no issues with multicollinearity; all values were well below the conventional threshold of 5 (range: 1.02–1.90, mean VIF = 1.67). To facilitate a direct comparison of effect sizes across dimensions, all continuous predictors were standardized (Z-scores) prior to analysis (Cohen et al., 2013).

The regression results are presented as follows. The standardized regression coefficients (*β*) and 95% confidence intervals of all AQ subscales are shown in Table 2.

**Table 2:** Intercorrelations among core variables, split by gender.

Variable	1	2	3	4	5	6	7
1. AQ Social Skills	—	0.48***	0.19***	0.39***	−0.32***	−0.13**	−0.18***
2. AQ Communication	0.38***	—	0.24***	0.31***	−0.17***	−0.18***	−0.21***
3. AQ Attention to Detail	0.13***	0.21***	—	−0.04	0.10**	0.08	0.10**
4. Loneliness	0.39***	0.29***	−0.06	—	−0.34***	−0.14**	−0.23***
5. Life satisfaction	−0.38***	−0.21***	0.14***	−0.40***	—	0.13**	0.25***
6. Personality similarity (fq6)	−0.15***	−0.10*	0.04	−0.17***	0.20***	—	0.41***
7. Interest similarity (fq7)	−0.16***	−0.19***	0.06	−0.26***	0.28***	0.40***	—

Correlations for males (*n* = 442) are presented above the diagonal; correlations for females (*n* = 630) are presented below the diagonal. Diagonal entries are dashes (—). AQ = Autism‑Spectrum Quotient. ***p < 0.001, **p < 0.01, *p < 0.05.

**Figure 1:**
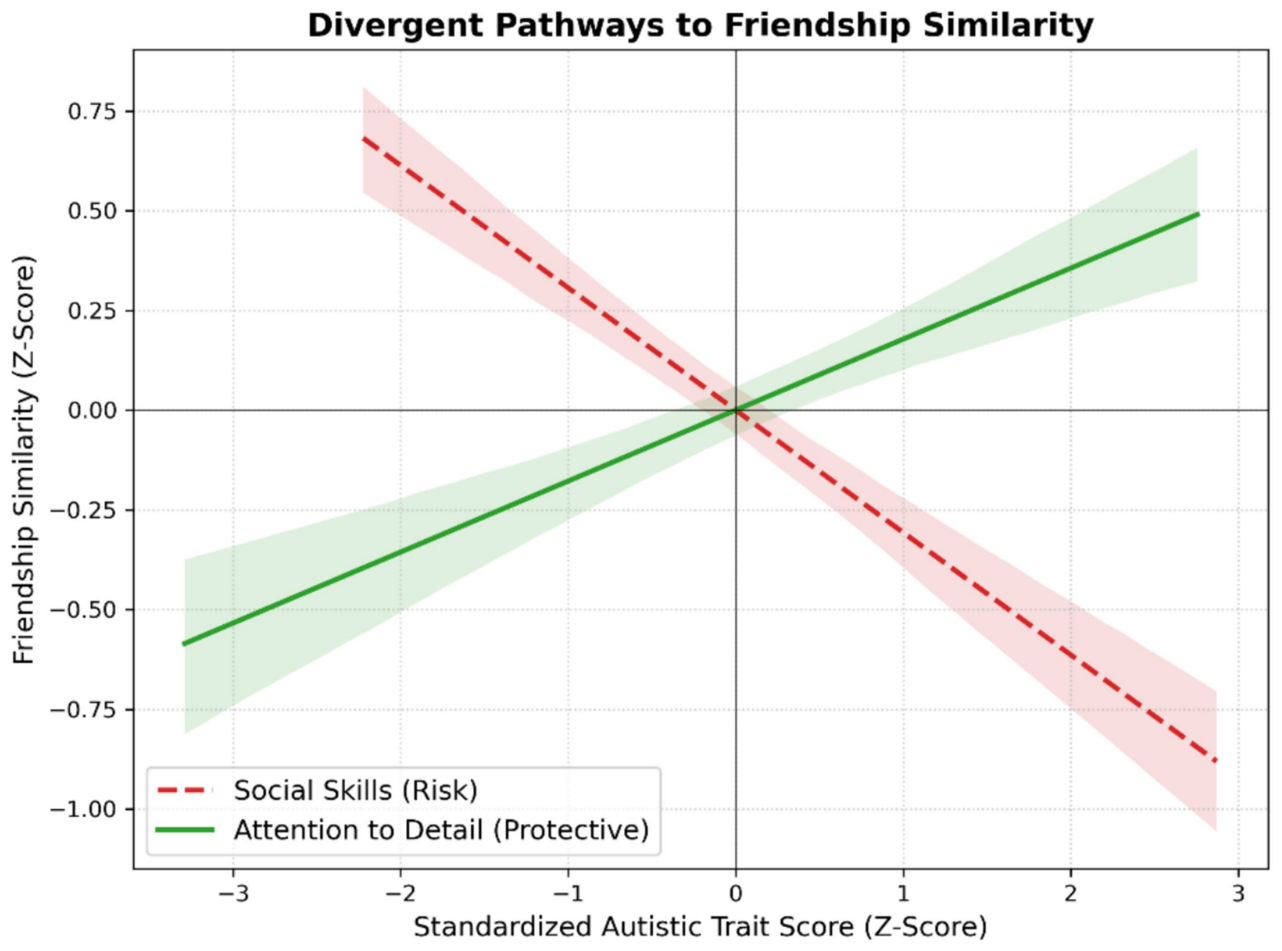
The “Double-Edged Sword” of the Broader Autism Phenotype: Opposing Associations of Social versus Cognitive Traits with Friendship Similarity Note. Marginal effects plot illustrating the opposing slopes of Social Skills (red dashed line) and Attention to Detail (green solid line) on friendship similarity. Shaded areas represent 95% confidence bands.

A marginal effects plot (Figure 1) was generated to illustrate the divergent slopes of the most significant social trait (Social Skills) and cognitive trait (Attention to Detail) against interest similarity, providing an immediate visual contrast between risk factors and protective factors.

Finally, a serial mediation analysis was performed to examine whether the divergent effects of AT dimensions on life satisfaction were mediated by interest similarity (fq7) and loneliness. Indirect effects were tested using 5,000 bootstrap samples to generate bias-corrected 95% confidence intervals, following the procedure recommended by Hayes (Hayes, 2017). Additionally, a robustness check was performed by substituting personality similarity (fq6) for interest similarity (fq7) as the mediator, to empirically test whether the protective pathway operates specifically through shared cognitive interests rather than through generalized interpersonal similarity.

## 3 Results

### 3.1 Descriptive statistics and correlations

Descriptive statistics for all core variables, stratified by gender, are presented in Table 3. The final analytical sample comprised 1,072 participants (630 females, 442 males) after excluding 4 participants who self-identified as other gender. The sample exhibited a normative distribution of total autistic traits (*M* = 118.18, SD = 12.13, range = 75–167). Consistent with prior research on the Friendship Questionnaire (Baron-Cohen and Wheelwright, 2003; Sedgewick et al., 2019), females reported significantly higher social skills difficulties than males (*M* = 2.39 vs. *M* = 2.22, *p* < 0.001, *d* = 0.31), whereas males reported significantly higher life satisfaction (*p* = 0.025, *d* = −0.14) and higher interest similarity with friends (*p* = 0.014, *d* = −0.15). No significant gender differences emerged on AQ Attention to Detail, Communication, or loneliness.

**Table 3:** Descriptive statistics of core variables by gender.

Variable	Total (*N* = 1,072)		Female (*n* = 630)		Male (*n* = 442)		*t*	*p*	*d*
	*M*	SD	*M*	SD	*M*	SD			
AQ Social Skills	2.32	0.55	2.39	0.57	2.22	0.51	4.95	< 0.001	0.31
AQ Communication	2.20	0.42	2.18	0.42	2.23	0.42	−1.91	0.057	−0.12
AQ Attention to Detail	2.61	0.40	2.60	0.41	2.62	0.38	−0.82	0.414	−0.05
Loneliness	2.04	0.68	2.07	0.68	1.99	0.69	1.93	0.054	0.12
Life satisfaction	4.56	1.30	4.48	1.30	4.66	1.29	−2.25	0.025	−0.14
Personality similarity (fq6)	2.80	0.70	2.79	0.72	2.81	0.69	−0.41	0.683	−0.03
Interest similarity (fq7)	2.97	0.78	2.93	0.79	3.05	0.78	−2.47	0.014	−0.15

*N* = 1,072 after excluding 4 participants who selected other gender. AQ = Autism‑Spectrum Quotient (1–4 Likert scoring). Loneliness measured by the UCLA Loneliness Scale short form (1–4 scale). Life satisfaction measured by the Satisfaction with Life Scale (1–7 scale). Friendship similarity items (fq6, fq7) scored 1–4. Cohen’s d: positive values indicate higher scores for females; negative values indicate higher scores for males. All t tests based on 1,070 df.

Table 2 displays the zero-order correlations separately for males (above the diagonal) and females (below the diagonal). The overall pattern of associations was highly consistent across genders. AQ Social Skills was significantly and negatively correlated with both personality similarity (*r* = −0.13 to −0.15, *p* < 0.01) and interest similarity (*r* = −0.16 to −0.18, *p* < 0.001). In contrast, AQ Attention to Detail demonstrated a modest positive correlation with interest similarity in males (*r* = 0.10, *p* < 0.01) and a non-significant positive correlation in females (*r* = 0.06, *p* = 0.13). Loneliness was significantly and negatively associated with life satisfaction in both genders (males: *r* = −0.34; females: *r* = −0.40; both *p* < 0.001).

### 3.2 Divergent predictors of interest similarity

A hierarchical multiple regression analysis was conducted to disentangle the unique contributions of social versus cognitive autistic traits to interest similarity (fq7). Demographic covariates (gender, age group, education, STEM discipline, and friendship status) were entered in Step 1, followed by the four AQ subscales in Step 2. All variance inflation factors (VIFs) were below 2.04, confirming that multicollinearity did not inflate the standard errors.

In Step 1, demographic covariates accounted for 5.0% of the variance (*R*^2^ = 0.050). Age group was the strongest predictor: participants aged 25–45 years reported significantly higher interest similarity than the younger reference group (*β* = 0.183, *p* < 0.001). Having a close friend was also a significant positive predictor (*β* = 0.097, *p* = 0.001), whereas gender, education, and STEM discipline were non-significant.

In Step 2, the addition of the AQ subscales significantly improved the model (Δ*R*^2^ = 0.062, *p* < 0.001), with the full model explaining 11.2% of the variance (*F*(9, 1,062) = 14.92, *p* < 0.001). As shown in Table 4 and visualized in Figure 1, the results supported the divergent pathways hypothesis:

- Social-communicative traits as risk factors. AQ Social Skills [*β* = −0.105, 95% CI (−0.186, −0.025), *p* = 0.011] and AQ Communication [*β* = −0.117, 95% CI (−0.196, −0.039), *p* = 0.004] were significant negative predictors of interest similarity. These findings indicate that greater social-communicative difficulties are associated with lower perceived similarity of interests with friends.
- Attention to Detail as a protective factor. AQ Attention to Detail was a positive predictor of interest similarity [*β* = 0.059, 95% CI (−0.001, 0.120), *p* = 0.055]. Although this coefficient fell just short of conventional significance in the full regression model after controlling for all covariates, its direction was consistent with the hypothesis and reached significance in the serial mediation model (see Section 3.3).
- Other cognitive features. The composite index of Attention Switching and Imagination did not significantly predict interest similarity (*β* = −0.052, *p* = 0.177).

**Table 4:** Hierarchical multiple regression predicting interest similarity (fq7).

Predictor	Step 1 *β*	Step 2 *β*	Step 2 95% CI	*p*
Step 1: Demographics				
Male	0.064	0.069	[0.008, 0.131]	0.027
Age (25–45 years)	0.183	0.117	[0.058, 0.177]	<0.001
Education (graduate)	0.005	−0.008	[−0.056, 0.040]	0.780
STEM discipline	−0.001	−0.008	[−0.069, 0.054]	0.805
Has close friend	0.097	0.045	[−0.012, 0.102]	0.125
Step 2: AQ subscales				
Social skills	—	−0.105	[−0.186, −0.025]	0.011
Communication	—	−0.117	[−0.196, −0.039]	0.004
Attention to detail	—	0.059	[−0.001, 0.120]	0.055
Other cognitive features	—	−0.052	[−0.127, 0.023]	0.177
*R* ^2^	0.050	0.112		
Δ*R*^2^	—	0.062		<0.001

*N* = 1,072. *β* = standardized regression coefficient from the full model (Step 2). CI = confidence interval. Δ*R*^2^ = change in *R*^2^ from Step 1 to Step 2. Other Cognitive Features = composite of Attention Switching and Imagination subscales (excluded from primary mediation analyses). All VIFs < 2.04. Due to differences in estimation methods, the path from Attention to Detail to fq7 reached significance in the simultaneous SEM framework (see Table 4).

It is worth noting that the coefficient for Attention to Detail in the hierarchical OLS regression fell just short of conventional significance (*β* = 0.059, *p* = 0.055). However, in the full serial mediation model estimated simultaneously via maximum likelihood (Table 5), the same path was significant (*B* = 0.012, *p* = 0.044). This slight discrepancy likely reflects the fact that SEM uses full-information estimation and simultaneously accounts for all direct and indirect covariances, which can yield greater statistical power for individual paths than the stepwise OLS framework. Given the theoretical centrality of this pathway and its significance in the full model, we interpret Attention to Detail as a meaningful predictor of interest similarity.

**Table 5:** Serial mediation model: path coefficients and indirect effects.

Panel A: path coefficients					
Path	*B*	*SE*	z	*p*	95% CI
Predicting interest similarity (fq7)					
Social Skills → fq7	−0.017	0.006	−3.10	0.002	[−0.028, −0.006]
Communication → fq7	−0.026	0.007	−3.65	<0.001	[−0.039, −0.012]
Attention to Detail → fq7	0.012	0.006	2.02	0.044	[0.000, 0.024]
Predicting loneliness					
fq7 → Loneliness	−0.112	0.024	−4.71	<0.001	[−0.158, −0.065]
Social Skills → Loneliness	0.038	0.004	8.81	<0.001	[0.030, 0.047]
Communication → Loneliness	0.029	0.005	5.23	<0.001	[0.018, 0.039]
Attention to Detail → Loneliness	−0.006	0.005	−1.24	0.214	[−0.015, 0.003]
Predicting life satisfaction					
fq7 → Life Satisfaction	0.198	0.045	4.42	<0.001	[0.110, 0.286]
Loneliness → Life Satisfaction	−0.429	0.057	−7.52	<0.001	[−0.541, −0.317]
Social Skills → Life Satisfaction	−0.070	0.008	−8.30	<0.001	[−0.087, −0.053]
Communication → Life Satisfaction	0.001	0.010	0.01	0.991	[−0.020, 0.020]
Attention to Detail → Life Satisfaction	0.018	0.009	2.01	0.044	[0.000, 0.035]

Panel B: indirect effects of AQ subscales on life satisfaction			
Indirect pathway	*B*	Bootstrap *SE*	BC 95% CI
Via interest similarity and loneliness (fq7)			
Social Skills → fq7 → Loneliness → Life Satisfaction	−0.00083	0.00039	[−0.00186, −0.00027]
Communication → fq7 → Loneliness → Life Satisfaction	−0.00123	0.00049	[−0.00250, −0.00051]
Attention to Detail → fq7 → Loneliness → Life Satisfaction	0.00059	0.00038	[0.00006, 0.00159]
Robustness check: via personality similarity (fq6)			
Social Skills → fq6 → Loneliness → Life Satisfaction	−0.00072	0.00038	[−0.00183, −0.00021]
Communication → fq6 → Loneliness → Life Satisfaction	−0.00072	0.00039	[−0.00176, −0.00015]
Attention to Detail → fq6 → Loneliness → Life Satisfaction	0.00037	0.00028	[−0.00002, 0.00114]

*N* = 1,072. Model controlled for gender, age, education, STEM discipline, and friendship status. *B* = unstandardized regression coefficient. BC 95% CI = bias‑corrected 95% confidence interval from 5,000 bootstrap resamples. Indirect effects in bold are statistically significant (BC 95% CI excludes zero). Unstandardized coefficients are reported because the AQ subscales use the original 1–4 Likert metric; multiplying the coefficient by 10 yields the approximate effect for a full scale‑point difference. The robustness check substitutes personality similarity (fq6) for interest similarity (fq7).

As a sensitivity check, we re-estimated the primary regression model excluding the ‘Other Cognitive Features’ composite. The pattern of results for Social Skills, Communication, and Attention to Detail remained unchanged, with the latter’s standardized coefficient remaining positive and marginally significant. This confirms that the inclusion of this low-reliability composite did not distort the focal estimates.

### 3.3 Serial mediation analysis

To examine whether the divergent effects of autistic trait dimensions on life satisfaction were mediated by interest similarity and loneliness, a serial mediation analysis was estimated with 5,000 bootstrap resamples, controlling for all demographic covariates. Path coefficients and indirect effects are summarized in Table 5.

#### 3.3.1 The mediation chain

After accounting for autistic traits and covariates, the hypothesized sequential pathway was fully supported. Interest similarity significantly and negatively predicted loneliness (*β* = −0.112, *p* < 0.001), which in turn was a significant negative predictor of life satisfaction (*β* = −0.429, *p* < 0.001). Interest similarity also retained a significant direct positive association with life satisfaction (*β* = 0.198, *p* < 0.001), indicating that perceived homophily contributes to well-being both directly and indirectly through reduced loneliness.

*Divergent indirect effects.* Bootstrap estimates confirmed opposing indirect pathways from distinct autistic traits to life satisfaction via the interest similarity–loneliness chain (Table 5, Panel B):

- *Risk pathway*. Both AQ Social Skills [indirect effect = −0.00083, bias-corrected 95% CI (−0.00186, −0.00027)] and AQ Communication [indirect effect = −0.00123, BC 95% CI (−0.00250, −0.00051)] exerted significant negative indirect effects on life satisfaction. These findings indicate that social-communicative difficulties are indirectly associated with lower life satisfaction by reducing interest similarity, which in turn predicts higher loneliness.
- *Protective pathway*. In contrast, AQ Attention to Detail exhibited a significant positive indirect effect on life satisfaction (indirect effect = 0.00059, BC 95% CI [0.00006, 0.00159]). Although numerically small, the confidence interval excludes zero, confirming that a detail-oriented cognitive style is indirectly associated with greater life satisfaction by facilitating interest-based friendship similarity and subsequently reducing loneliness.

#### 3.3.2 Robustness check with personality similarity

When personality similarity (fq6) replaced interest similarity as the mediator, the negative indirect effects of Social Skills [indirect effect = −0.00072, BC 95% CI (−0.00183, −0.00021)] and Communication [indirect effect = −0.00072, BC 95% CI (−0.00176, −0.00015)] remained significant. However, the positive indirect effect of Attention to Detail was not significant [indirect effect = 0.00037, BC 95% CI (−0.00002, 0.00114)]. This divergence suggests that the protective pathway operates specifically through shared cognitive interests rather than through generalized perceptions of personality compatibility.

## 4 Discussion

The present study utilized a serial mediation framework to deconstruct the heterogeneous pathways through which distinct dimensions of autistic traits (ATs) predict loneliness and life satisfaction among Chinese adults. By shifting the empirical focus from a monolithic deficit lens to a multidimensional, strengths-based paradigm, our findings substantiate a clear bifurcation within the Broader Autism Phenotype (BAP). Specifically, social-communicative difficulties (Social Skills and Communication) exhibited a robust risk-driven pathway to loneliness by hindering perceived interest similarity with friends, which in turn predicted lower life satisfaction. In sharp contrast, the cognitive trait of Attention to Detail emerged as an adaptive asset: it was indirectly associated with higher life satisfaction through the same chain of interest similarity and reduced loneliness. This dual-pathway mechanism provides critical empirical support for the Double Empathy framework (Milton, 2012) within a non-Western collectivist culture, highlighting that neurodivergent social connection operates through relational resonance rather than absolute deficit.

### 4.1 Cultural nuances in measuring autistic traits: reconsidering the role of attention to detail

The present findings indicate that the Attention to Detail dimension of the Autism Spectrum Quotient operates differently from social-communicative traits in the Chinese context. While social skills and communication difficulties were associated with lower perceived similarity in friendships, Attention to Detail showed a positive association with interest similarity—an association that ultimately translated into a significant positive indirect effect on life satisfaction [indirect effect = 0.00059, BC 95% CI (0.00006, 0.00159)].

This pattern is consistent with previous psychometric studies conducted in East Asian populations, which have reported that Attention to Detail often exhibits weaker loadings on a general autism factor and shows distinct correlational patterns compared to social and communication subscales (Ward et al., 2021; Zhang et al., 2016).

In the Chinese cultural context, which places a high value on academic diligence, scholarship, and technical precision, the cognitive tendency to focus on details may be reframed as a strength rather than a deficit (Zhao et al., 2020). Unlike social rigidity, which conflicts with the collective mandate for interpersonal harmony (guanxi), a detail-oriented cognitive style is often rewarded in educational and professional settings (Meng and Xuan, 2023). Our data extends this observation to the social domain: Attention to Detail was the only AQ subscale to positively predict interest similarity, and this association held after controlling for demographic characteristics, STEM discipline, and friendship status. This supports the argument that the BAP structure is culturally sensitive, and future research in non-Western samples should exercise caution when using total AQ scores, as they may mask the adaptive potential of specific cognitive dimensions (Chee and De Vries, 2021).

### 4.2 Beyond quantity: friendship similarity and the experience of loneliness

A central implication of the present findings is that the subjective quality of social relationships, as indexed by perceived similarity, plays a more critical role in loneliness than the mere presence of friendships. Although 93.7% of participants reported having at least one close friends, higher levels of social autistic traits were still associated with elevated loneliness. This indicates that relational availability alone is insufficient to alleviate feelings of social disconnection. This pattern is consistent with prior research in individuals with elevated autistic traits, who may maintain social ties yet continue to experience a sense of isolation (Feller et al., 2024; Płatos and Pisula, 2021). Our supplementary model further suggested that interest similarity may help sustain the presence of a close friendship, which in turn buffers against loneliness. However, the direct path from interest similarity to loneliness remained significant, confirming that perceived homophily operates primarily through subjective relational quality rather than mere relationship presence.

The current results provide empirical support for relational mismatch perspectives, including the Double Empathy framework (Milton, 2012), which conceptualizes social difficulty as arising from mutual misunderstanding between individuals with differing communicative styles rather than from unilateral social difficulties (Galvin et al., 2024; Milton, 2012). From this perspective, loneliness is not simply a function of limited social contact but reflects a lack of interpersonal resonance (Jobe and White, 2007; Stice and Lavner, 2019). Friendships characterized by low perceived similarity may fail to provide emotional affirmation, shared understanding, or a sense of mutual attunement, even when they are structurally present (Suzuki et al., 2021).

In this regard, perceived interest similarity appears to function as a key relational resource. When individuals perceive their friends as sharing similar interests, interactions may require less effortful adjustment and social monitoring (Cresswell et al., 2019). This may be particularly salient for individuals with elevated autistic traits, for whom navigating implicit social norms and conversational subtleties can be cognitively demanding (Benford and Standen, 2009; Lamport and Zlomke, 2014). Interest similarity-based friendships may therefore reduce the experiential distance between self and other, fostering a sense of being understood rather than merely included. To contextualize these dynamics within a broader theoretical landscape, the social connection framework (Holt-Lunstad, 2022, 2025) conceptualizes social health as a complex ecosystem rather than a binary state of deficit. Within this architecture, the mitigation of loneliness does not rely exclusively on intensive, high quality dyadic relationships; rather, the value of “weak ties” and incidental social contacts—such as those found in subcultural niche interactions—serves as a critical buffer for individuals with high autistic traits (Pellicano and Heyworth, 2025). Future research should examine whether interest based communities (e.g., online forums, hobby groups) provide alternative relational niches that reduce loneliness without requiring intensive dyadic friendship.

### 4.3 The risk pathway: social-communicative traits, relational mismatch, and well-being

Consistent with existing literature, social skills and communication difficulties emerged as significant negative predictors of interest similarity, which in turn was associated with higher loneliness and lower life satisfaction (Mazurek, 2014). The bootstrap confirmed indirect effects (Social Skills: −0.00083; Communication: −0.00123) underscore the central role of social communicative functioning in shaping the subjective quality of interpersonal relationships.

In Chinese sociocultural context, these dynamics may be further intensified. Social competence is often closely tied to moral evaluation and social credibility, and difficulties in interpersonal engagement may be interpreted as a lack of sincerity, adaptability, or emotional sensitivity (Lau et al., 2013; Zhao et al., 2020). Such interpretations can constrain opportunities for meaningful connection and may contribute to a cycle in which social challenges lead to reduced relational quality, reinforcing feelings of exclusion and loneliness (Kang et al., 2020). Over time, this pattern may have cumulative implications for subjective well-being.

### 4.4 The protective pathway: attention to detail and the formation of similarity-based friendships

One of the more distinctive patterns observed in the present study is the positive association between Attention to Detail on life satisfaction through the interest similarity–loneliness chain. Although the point estimate is numerically small (0.00059), its bias corrected bootstrap 95% confidence interval excludes zero [0.00006, 0.00159], confirming statistical reliability. Rather than dismissing this as a trivial effect, we interpret its modest magnitude as indicative of an “ecological niche effect” (Baron-Cohen, 2006).

Grounded in Systemizing Theory, a detail oriented cognitive style does not serve as a panacea for global social integration; instead, its adaptive utility is context dependent, yielding its greatest returns within structured, predictable, and rule bound relational niches—such as STEM fields, academic environments, or highly specialized hobbyist domains. By aligning communication around shared, objective interests rather than ambiguous socio emotional cues, this cognitive style provides a structured communication anchor that lowers cognitive load and reduces the need for exhaustive social camouflaging (Liu et al., 2024). Within these niches, shared epistemic interests become the foundational currency for friendship similarity, proving that even a subtle cognitive asset can emit a resilient protective signal against loneliness. Critically, our robustness check distinguishes the mechanism underlying this protective pathway. When personality similarity (fq6) replaced interest similarity as the mediator, the positive indirect effect of Attention to Detail was not significant (indirect effect = 0.00037, BC 95% CI [−0.00002, 0.00114]). This divergence indicates that the protective pathway operates specifically through shared cognitive interests—such as mutual fascination with specific topics, technical hobbies, or analytical pursuits—rather than through generalized perceptions of personality compatibility. This specificity aligns with the assortative affiliation hypothesis: individuals with a detail oriented cognitive style may preferentially seek out peers who share similar analytical processing styles, thereby forming friendships based on shared systemizing interests that minimize social unpredictability. From a sociological perspective, the similarity-based friendships observed in our Chinese sample do not merely represent traditional normative conformity. In a collectivist society where mainstream sociality is heavily moralized and deeply embedded in hierarchical guanxi networks, communication challenges are frequently penalized as a lack of social maturity. Within this rigid matrix, the interest-based friendships facilitated by Attention to Detail likely reflect the cultivation of subcultural niches—such as digital communities, anime/comic/game circles, or specialized technical groups. These subcultural spaces allow individuals with elevated autistic traits to bypass traditional, hierarchy driven social demands and forge lateral connections based on shared cognitive resonance, thereby carving out a sanctuary that buffers against mainstream social exclusion. Taken together, these results highlight the need to move beyond deficit oriented interpretations of autistic traits and to consider how different trait dimensions interact with social context to shape relational outcomes. The identification of Attention to Detail as a facilitator of interest based friendship underscores the heterogeneity of interpersonal pathways within the BAP.

### 4.5 Gender differences

Our descriptive analyses revealed several gender differences worthy of note. Consistent with prior research on the Friendship Questionnaire (Baron-Cohen and Wheelwright, 2003; Sedgewick et al., 2019), females reported significantly higher social skills difficulties than males, whereas males reported higher interest similarity with friends and higher life satisfaction. Gender was included as a covariate in all regression and mediation models to control for its potential confounding influence. We did not formally test for moderation of the indirect pathways by gender, and thus no conclusions regarding differential pathway strengths across genders can be drawn. Future research employing multi group or interaction based moderation analyses would be necessary to examine whether the protective and risk pathways operate differently for males and females, and employing separate measures of sex assigned at birth and gender identity, as recommended by the SAGER guidelines (Heidari et al., 2016), would be better positioned to investigate whether the protective pathway operates differently across sex and gender groups.

### 4.6 Limitations and future directions

Despite the insights provided by this study, several limitations must be acknowledged.

First, although we focused on the Chinese cultural context, our sample was recruited primarily through online social media platforms, which may have introduced a selection bias favoring individuals who are digitally active. Furthermore, we did not directly measure cultural values as a moderator. Future cross-cultural comparative studies would be valuable to definitively test whether the protective role of cognitive autistic traits is specific to East Asian contexts.

Second, the gender item used in this study captured self reported identity via a single question and did not separately assess biological sex (assigned at birth). So our sample included only four participants who self identified outside the gender binary, precluding separate analysis. Future research should purposively recruit gender diverse and non binary neurodivergent individuals, as their relational experiences may differ qualitatively from those captured by the binary gender framework used here.

Third, the structural design of the AQ, specifically its mixture of positively and reverse-scored items may introduce method variance and contribute to sub-optimal subscale reliabilities in non-clinical samples (English et al., 2020). In the present study, the Attention Switching and Imagination subscales exhibited poor internal consistency and were excluded from primary analyses, and the Attention to Detail subscale showed marginal reliability. Future work should consider alternative measures of detail-oriented cognition that offer stronger psychometric properties in general population samples. Finally, our use of two single-item measures for friendship similarity, while theoretically justified and empirically supported by the differential results for interest versus personality similarity, limits the depth with which these constructs were assessed. Multi-item scales capturing interest-based and personality-based homophily would strengthen future investigations.

## 5 Conclusion

This study provides comprehensive empirical evidence distinguishing the divergent pathways of social versus cognitive autistic traits on well-being in a Chinese adult sample. By deconstructing the Broader Autism Phenotype, we revealed a “double-edged sword” effect: while social-communicative difficulties act as a risk factor by eroding interest-based friendship similarity and fueling loneliness, the cognitive trait of Attention to Detail serves as a unique protective factor, indirectly supporting life satisfaction by facilitating shared interests that buffer against isolation. These findings challenge the monolithic deficit model of autism and underscore the importance of interest-based friendship morphology as a critical relational resource Ultimately, supporting the well-being of individuals with high autistic traits requires looking beyond their social difficulties to recognize and cultivate their cognitive strengths, fostering social environments, whether in person or in subcultural niches, where different does not mean disconnected (Caron et al., 2021).

## Data Availability

The raw data supporting the conclusions of this article will be made available by the authors, without undue reservation.
